# Investigation of the Behavioral Characteristics of Dogs Purpose-Bred and Prepared to Perform *Vapor Wake*^®^ Detection of Person-Borne Explosives

**DOI:** 10.3389/fvets.2018.00050

**Published:** 2018-03-20

**Authors:** Lucia Lazarowski, Pamela Sue Haney, Jeanne Brock, Terry Fischer, Bart Rogers, Craig Angle, Jeffrey S. Katz, L. Paul Waggoner

**Affiliations:** ^1^Canine Performance Sciences Program, College of Veterinary Medicine, Auburn University, Auburn, AL, United States; ^2^Department of Psychology, College of Liberal Arts, Auburn University, Auburn, AL, United States

**Keywords:** *Vapor Wake*^®^, detection dog, phenotype, behavior, selective breeding, working dogs, canine, person-borne explosives

## Abstract

Specialized detector dogs are increasingly being utilized for the detection of modern threats. The *Vapor Wake*^®^ (VW) dog was developed to create a dog phenotype ideally suited for detecting hand-carried and body-worn explosives. VW dogs (VWDs) are trained to sample and alert to target odors in the aerodynamic wakes of moving persons, which entrains vapor and small particles from the person. The behavioral characteristics necessary for dogs to be successfully trained and employed for the application of VW are a distinct subset of the desired general characteristics of dogs used for detection tasks due to the dynamic nature of moving targets. The purpose of this study was to examine the behavioral characteristics of candidate detector dogs to determine the particular qualities that set apart VW-capable dogs from others. We assessed 146 candidate detector dogs from a VW breeding and training program. Dogs received identical puppy development and foundational odor training and underwent performance evaluations at 3, 6, 10, and 12 months old, after which they were sold for service. Dogs were categorized based on their final outcome of the training program, independently determined by private vendors, corresponding to three groups: dogs successfully sold for VW, dogs sold for standard explosives detection, and dogs that failed to be placed in any type of detector dog service (Washouts). Comparisons of behavioral evaluations between the groups were made across domains pertaining to search-related behaviors (Performance), reactions to novel stimuli (Environmental), and overall ease of learning new tasks (Trainability). Comparisons were also made at each evaluation to determine any early emergence of differences. VWDs scored significantly higher on Performance characteristics compared to standard explosives detection dogs (EDDs) and Washouts. However, Environmental characteristics did not differentiate VWDs from EDDs, though scores on these measures were significantly lower in the Washouts. Furthermore, differences between groups emerged as early as 3 and 6 months for select measures. We describe the behavioral characteristics targeted for selection in developing the VW phenotype and discuss the relative merit and degree of expression of those characteristics in the success of dogs bred and trained for the VW application.

## Introduction

Detector dog applications are becoming ever more technically specialized. Examples of such specialization include the following: military off-lead, directionally controlled down-range improvised explosive device detection; cargo inspection; evidence retrieval; concealed human detection; pest and agricultural pathogen detection; and air passenger screening. The required characteristics of dogs for traditional detection tasks are also being more narrowly defined as state-of-the-art for certain applications, e.g., refining urban vs. wilderness search and rescue (SAR), immediate vs. aged human trail tacking, and trace vs. bulk substance detection. Growing recognition of canine olfaction as the most capable tool for the majority of detection tasks and growing technical sophistication of detector dog practitioners have given rise to the expansion of the types and specialization of detector dog applications. Consequently, the numbers of dogs exhibiting suitable characteristics to perform contemporary detector dog tasks have declined. Moreover, despite the widespread recognition of the important role of detector dogs in security operations, systematic examinations of the characteristics of such specialty search dogs are scarce in the literature (1–3). Additionally, there is a lack of standardization and consistency in identifying and describing specific desired detection dog behavioral characteristics and screening processes (1).

A primary means by which detector dogs are sourced is the selection of dogs from populations bred for purposes other than security-related detection tasks. An example of this repurposing of dogs is the selection of sporting breed dogs purpose-bred for hunting and field trial activities to be trained to perform detection tasks. With few notable, but fairly exclusive, exceptions, such as the Norwegian People’s Aid Global Training Centre for mine detection dogs selective breeding program (4), and the former Transportation Security Administration’s Canine Breeding Program for detection dogs, there have been only small-scale and short-lived efforts to breed dogs for specific detector dog applications. There are scant examples of technical or scientific reporting of such efforts, thus, there exists little formal research or technical guidance to provide direction in selective breeding of detector dogs (2, 5).

It is becoming increasingly acknowledged that behavioral characteristics are greater determinants of detector dog success than sensory or morphological characteristics (6, 7). Thus, accurately evaluating behavioral characteristics for selection and prediction of successful working dogs is vital for the sustainability of working dog programs. Maejima et al. (8) reported a 30% success rate of 197 Labrador retriever dogs entering drug detection programs and Wilsson and Sundgren (9) reported a 4.9% composite success rate for search tasks from 2,107 candidate German shepherd and Labrador retriever dogs. Given the low levels of successful candidate detector dogs reported across working dog programs, identifying and selecting for traits related to success as a detector dog are clearly challenging. Without the ability to identify the key behavioral characteristics that are predictive of successful candidate working dogs, precision in mating selection is greatly reduced, impeding advancement of specific capabilities in working dog populations.

It can be argued that traditional means of producing and raising most detector dogs are inadequate to meet the growing demand for specialized applications. One such specialized application that has emerged in response to modern threats, such as person-borne improvised explosives devices, is the *Vapor Wake*^®^ (VW) detection methodology (10, 11). VW detection dogs are trained to sample and alert to target odors in the aerodynamic wakes of moving persons, which entrains vapor and small particles from the person. The behavioral characteristics exhibited by dogs capable of performing VW detection differ from those of traditional standard explosive detector dogs (EDDs) that are trained to detect static odor sources. Vapor Wake dogs (VWDs) must independently and constantly sample the air making efficient use of air currents to interrogate the human aerodynamic wake for target odors (12, 13). VWDs must be highly vigilant in searching for target odors and resilient from distraction in high-stimulus environments, such as large event venues and mass transit stations, where they are most often utilized. Thus, the VW application requires dogs with a pronounced expression of what are generally considered desirable characteristics in all detection dogs, plus some distinct characteristics such as vigilance (i.e., sustained attention) in searching for and alerting to target odors and deference for engaging in such searching as compared to engaging in other activities, such as, particularly, social interaction with people. With the demand for VWDs rapidly growing due to increasing incidents of terrorism involving body-worn and hand-carried moving targets, identifying and characterizing these traits are critical to the successful application of VW technology.

The purpose of this study was to examine the degree of expression of behavioral characteristics traditionally associated with detector dogs capable of performing the VW task in comparison to dogs not capable of performing VW within the recent (i.e., since 2013) population of dogs produced by the Canine Performance Sciences (CPS) program within Auburn University’s College of Veterinary Medicine (AUCVM). To do so, performance evaluations of the purpose-bred population of candidate VWDs, performed at 3, 6, 10, and 12 months by CPS senior trainers, were compared between groups of dogs categorized according to their final disposition, i.e., whether the dog was ultimately sold as VWD, as an EDD, or failed to be sold as either (Washout), as determined by third-party customer independent evaluations. Additionally, comparisons between groups at each evaluation timepoint were conducted to determine whether early differences emerged, for the purpose of improvements in early identification of successful or unsuccessful candidates. We hypothesized that due to the rigorous demands imposed by performing VW, dogs qualifying for VW roles would exceed others in their behavioral and task-related performance characteristics. This work represents the first examination of behavioral characteristics and their accentuation through selective breeding and controlled early experiences that are related to the success of dogs in performing dynamic (i.e., moving persons) person-borne improvised explosive device detection.

## Materials and Methods

### Subjects

The original breeding stock from which dogs described in this paper have been bred came from Australian Customs Service in the year 2000. The initial goal of the CPS program with this original population was to breed high-quality detector dogs. Since then, American Field Trial, Hunt Test, and Upland Game dogs have been integrated into the breeding population to incorporate new genetics and diversify the population. Currently, CPS has bred a total of 121 litters of purpose-bred detection dogs. In 2013, CPS began a concerted effort using evaluation measures to specifically enhance traits that were thought to be particularly important for the VW application. Dogs selected as breeders must have superior detection and behavioral characteristics and no medical issues. Prior to the concerted effort in 2013, 70 + dogs had been previously sold as VWDs. Since 2013 until now, 38 litters of dogs have been purpose-bred for producing dogs capable of performing the VW application. This paper describes dogs (*n* = 146) bred and trained at the AUCVM CPS from the time this concerted effort began (September 2013) to September 2016. The sample consisted of 28 litters from 17 dams and 18 sires. A total of 9 dams and 8 sires were bred more than once. No sire and dam breeding matchings were repeated. Dogs were Labrador retrievers (*n* = 119) and Labrador retriever X German wirehaired pointer (GWP) crosses (*n* = 27; 11 of which were 50% GWP and the remaining 25% or less). The sample consisted of male (*n* = 71) and female (*n* = 75) dogs that remained intact until matriculation out of the breeding puppy development program. Dogs that were medically disqualified from service (*n* = 11) were not included in the analyzed sample (i.e., 146 dogs remained after removing 11 medically disqualified dogs). Medical disqualifications were due to orthopedic issues: hip dysplasia (*n* = 4), elbow dysplasia (*n* = 3), stifle issues (*n* = 2), hip and elbow dysplasia (*n* = 1), and an indeterminate biomechanical issue (*n* = 1). All dogs were born, reared, and housed in the same environment and participated in the same standard CPS development and training protocols, described below, from the time they were born until they were sold. Dog care and use activities were approved and monitored by the Auburn University Institutional Animal Care and Use Committee.

### CPS Puppy Development Phases

All dogs participated in standard CPS training and development protocols, intended to produce VW-quality dogs. CPS production and puppy development consists of 6 phases. In Phase 1, sires and dams are selected through a screening process for medical soundness, low inbreeding coefficients, and superior performance and environmental behavioral characteristics. Phase 2 consists of the breeding, gestation, and partition periods. In Phase 3, puppies are group-housed in the nursery with their littermates and mother until 7 weeks of age. This period of early puppy development includes the introduction of new sights and sounds, reward value building, and obstacle navigations to enhance motor skills and problem-solving abilities. In Phase 4, intermediate puppy development occurs through extensive social, environmental, and performance conditioning in Auburn, AL, USA and the surrounding areas and lasts from 7 weeks to 6 months of age. Puppies are housed in indoor/outdoor kennels, first pair-housed until 13 weeks and then single-housed. Successive approximation of age-appropriate conditioning and exposures, progressing from simple to complex using positive reinforcement, is used to cultivate a strong foundation for detector dog training. Intermediate puppy development continues through Phase 5 when at 6 months puppies are placed in participating prisons for further socialization and development by specially trained inmates until 10 months of age. Inmates participating in the program are enrolled in a 1,150-h CPS-developed Performance Canine Care and Development course taught in the prisons by trained program managers. The prison program engages dogs in activities like basic odor discrimination games and exposes dogs to tighter living quarters simulating operational work in crowds of people. Phase 6, final puppy development, commences upon return from the prison program at 10 months of age until 12 months of age. During this 2-month period, dogs undergo evaluations for detection performance, physical fitness, environmental soundness (i.e., responsivity to environmental stimuli), and medical soundness. Dogs receive 16 days of VW foundational training, undergo final behavioral evaluations, and complete their puppy development cycle at CPS by final placement through sale as a VWD or EDD, retained for CPS breeding or research activities, or, infrequently, offered for adoption.

### Behavioral Evaluations

Evaluations were conducted by expert observers when the dogs were 3, 6, 10, and 12 months old. Evaluations consisted of 14 measures across three domains: seven Performance measures, six Environmental measures, and one overall Trainability measure. Performance measures consisted of characteristics associated with detection and searching abilities. Behaviors underlying a dogs’ motivation to search are commonly collectively referred to as a dogs’ “drive,” or a natural motivation to perform a particular action. Several types of drives important to detection dog success have been described in the literature, including play drive (a dogs’ desire to entertain itself by engaging with others or objects), prey drive (desire to chase and kill), and hunt drive (dogs’ desire to search for hidden prey using their nose) (1, 14). Environmental measures consisted of responses and reactions to unfamiliar stimuli in the environment. Sometimes referred to as “nerve strength,” these measures largely focus on the dogs’ ability to deal with and adapt to stress-inducing experiences, and include tactile, auditory, and visual stimuli (15). Finally, Trainability consisted of just one measure of a dogs’ ease and speed of learning new tasks (1). Table 1 contains detailed descriptions of each item within each domain. These domains and evaluated characteristics are commonly used in the assessment of candidate detector dogs in the working dog industry that, over time, CPS has tailored and operationally defined for use in assessing the potential of dogs for successfully performing VW detection. Each characteristic assessed has a defined “most desirable” expression that should engender a score of 5, on a 1–5 Likert scale. The most desired expression of some characteristics are multifaceted and not a unidirectional, less-to-more display of a particular response, but rather the extent to which the expression of a, sometimes complex, pattern of behavior in response to particular stimuli in a particular context has, in the program’s experience, been indicative of success as a VWD.

**Table 1:** Descriptions of measures assessed during performance evaluations, scored on a 1–5 scale from least to most desirable performance.

Domain	Measure	Definition
Performance	Retrieve	Dog will enthusiastically retrieve any reward every time with full sprints out and back
	Hunt	Dog constantly uses nose to search and investigate targets using closed-mouth search, not looking for handler guidance. Dog does not become over-excited when target odor is present and does not get discouraged when odor is not easily found
	Focus	Dog is able to focus on rewards/tasks. Dog notices environmental stimuli, but does not respond to distractions (i.e., urine, ambient noises)
	Physical possession	Dog holds reward in mouth, returns to handler holding reward, and looks for engagement with handler
	Independence	Dog is willing to work at a distance from handler and spends a minimum amount of time looking back for assistance
	Work effort	Dog will give 100% effort on every search/task every time. Dog is eager to find target to interact with handler
	Air scenting	Dog is constantly using nose to find air currents, while consistently and efficiently searching air. Dog is not looking at specific targets/objects

Environmental	Surfaces	Dog will transition across any and all kinds of surfaces without any hesitation
	People	Dog notices people, but does not try to interact. Dog may sniff people, but does not focus on people. Does not show fear, distraction, or excitement elicited by people
	Vehicles/urban clutter	Dog adapts to clutter and works normally without disruption in searching behavior. The urban clutter should elicit the dog’s searching behavior
	Visual startle	Dog notices new, unusual, or sudden stimuli but quickly resumes working. Dog may react by noticing stimuli, but holds ground and recovers quickly and then goes forward to investigate area
	Acoustic startle	Dog will notice loud stimuli, but holds ground and recovers quickly and then goes forward to investigate area
	Excitability	Dog is very active, exited to work, but not erratic. Dog may run through odor, but can recover and return to scent cone without giving up on task

General	Trainability	Dog is easily trainable. Dog learns new tasks quickly and easily with few trials and little direction

*The descriptions listed above reflect the standard VWD*.

Each evaluation was conducted over two consecutive days. All evaluations had portions consisting of both on- and off-lead tasks simulating real-world detection scenarios. Evaluations were tailored to be appropriate for each age level. Scoring used a subjective 5-point Likert scale with higher scorers indicating more desirable performance. Observers were senior canine instructors at CPS ranging from 8 to 35 years of experience in the handling and training of detector dogs in operational environments. At least one and up to three of the same three observers evaluated each dog; 68% of the observations had two or more evaluators. These instructors did not directly participate in the activities of raising and preparing the dogs for detection training from 0 to 10 months of age. At final training at 10 months, one or more may have been involved in the advanced detector dog training. The intent was to always have at least one evaluator that had not participated in the dogs’ training, which was most often the case.

### Final Disposition Categories

After completion of the CPS puppy development and training cycle, each dog was assigned a final disposition category based on its placement in service, which was determined independently by third-party customers. The goal of the CPS breeding program is that all dogs are placed in service as VWD; those not accepted for VW service are offered for service as an EDD, or, having been assessed as not suitable for service as either, retained for CPS research or prison teaching assistant dogs. Infrequently, dogs not suitable for sale were adopted out as a pet.

Aside from deciding which dogs to present to vendors as VWD/EDD candidates or withhold for presentation, CPS personnel were not involved in customers’ assessment or purchase decisions. Trainers’ filtering of which dogs to present to vendors is a practical matter of not presenting dogs that are demonstrably incapable of performing VW. There is strong program performance and financial motivation for CPS to present all dogs with even marginal chances of being selected for service to customers.

Upon initial presentation, the customer performs a series of performance and environmental tests in environments unfamiliar to the dogs to assess their potential for VW. At this point, a dog may be rejected as VW and downgraded to EDD or assessed as not suitable for detector dog work by the VW customer. Furthermore, the customer has a 30-day period in which they engage dogs in training in which to reject or accept the dog as VWD or EDD. Dogs returned to CPS by the customer within this window are further assessed by CPS for their potential to be sold as EDD to other, non-VW, customers. Dogs that CPS trainers assess as being demonstrably incapable of performing VW but may have potential as EDD are also presented to these other, non-VW customers. Dogs presented to those other customers, again, are subjected to independent assessment regimens of those programs and their final disposition is determined by whether those dogs are accepted (i.e., purchased) by those customers. Therefore, while the final dispositions of dogs in this study are not entirely independent due to trainer selection of which dogs to present to customers, there is significant practical pressure on CPS to present all dogs with the possibility of being sold as VWD or EDD to independent assessment for their operational detector dog capability, which determined their final disposition for the purpose of analysis in this study. This is a real-world scenario that adds significant ecological validity to the final outcomes observed in this study. Thus, for purpose of analysis in this paper, a dog’s final disposition was categorized as having been successfully placed in service as a VWD (and retained beyond the 30-day return window), EDD, or, if not selected for service, as a Washout. Dogs selected as breeders were also characterized as VWD (breeders are subsequently sold as VWD after completion of breeding, unless they are unable to be sold due to age). Washout dogs were further categorized as having failed due to inadequate performance, environmental soundness, or both. It is important to note that all dogs in the population were trained for the same goal of sale as a VWD, and to this end experienced the same training. Group categorizations as VWD, EDD, or Washout were made *post hoc* according to their sale status; dogs’ training or other experiences prior to sale did not differ.

### Data Analysis

Evaluators’ scores for each item were averaged to create a single score for each measure for each dog. Average scores for each group were compared for each of the items at each evaluation timepoint. Additionally, timepoints were collapsed and items pertaining to the same domain were averaged in order to create composite Performance, Environmental, and Trainability scores for each dog. Some dogs were not available for all evaluations or at one or more timepoints and thus were not included in certain analyses that excluded missing cases. Additionally, some items were more recently developed and thus scores for earlier dogs were not available. Measures that did not include all dogs were: Retrieve, Focus, and Work Effort at 10 mo (*n* = 141) and Final (*n* = 133); Hunt and Independence at 10 mo (*n* = 141) and Final (*n* = 132); Possession at 3 mo (*n* = 123), 6 mo (*n* = 137), 10 mo (*n* = 141), and Final (*n* = 132); Air Scenting at 3 mo (*n* = 110), 6 mo (*n* = 115), 10 mo (*n* = 126), and Final (*n* = 131); Surfaces, People, and Vehicles at 6 mo (*n* = 137) and Final (*n* = 136); Visual Startle at 10 mo (*n* = 136) and Final (*n* = 130); Acoustic Startle at 10 mo (*n* = 139) and Final (*n* = 130); Trainability at 10 mo (*n* = 144) and Final (*n* = 133); and Excitability at 3 mo (*n* = 92), 6 mo (*n* = 15), 10 mo (*n* = 128), and Final (*n* = 131). All analyses used IBM SPSS Statistics version 23 and an alpha level of .05.

## Results

### Final Disposition

The final dispositions of 146 CPS-produced dogs (after removal of 11 medical releases) were 63% VWD, 17% EDD, and 20% Washouts. Of the Washouts, 62.5% failed for insufficient environmental soundness and 37.5% failed for inadequate performance.

### Behavioral Evaluations

**Figure 1:**
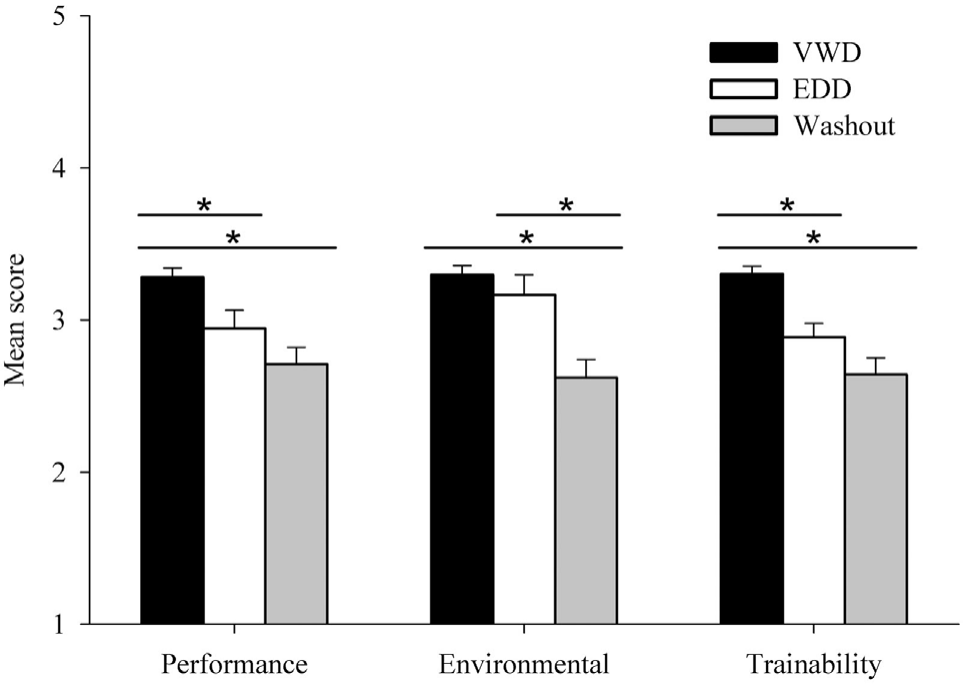
Average scores for each group [VW dog (VWD), explosives detection dog (EDD), Washout] in the Performance, Environmental, and Trainability domains. Mean scores represent averages of submeasures corresponding to each domain and are collapsed across timepoints. Error bars represent standard errors of the means. *p < 0.05.

Composite Performance, Environmental, and Trainability scores for each of the three final disposition groups (VWD, EDD, Washout) were calculated by averaging all component measures of the corresponding evaluative domain across the four timepoints (Figure 1). Separate one-way analysis of variance (ANOVA) for Group (VWD, EDD, Washout) were conducted for composite Performance, Environmental, and Trainability mean scores and all yielded a significant effect, *F*’s (2, 143) > 12, *p*s < 0.01. The VWD group outperformed the other groups in each domain except for Environmental where VWD and EDD were equivalent (see Figure 1), as confirmed by *post hoc t*-test comparisons.

### Performance Domain

**Figure 2:**
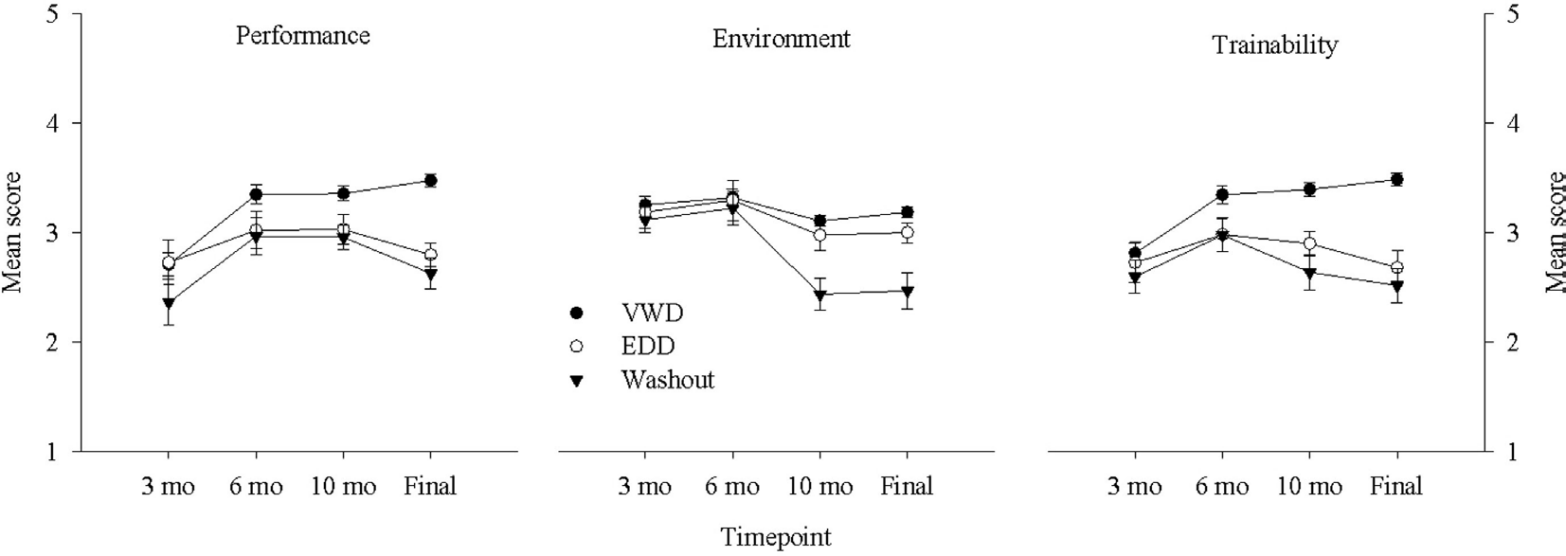
Average scores for each group [VW dog (VWD), explosives detection dog (EDD), Washout] in the Performance (left panel), Environmental (middle panel), and Trainability (right panel) domains across each of the evaluation timepoints (3 mo, 6 mo, 10 mo, and Final Evaluation). Error bars represent standard errors of the means.

Figure 2 (left panel) shows the composite mean score for all Performance measures across the four timepoints. A two-way repeated-measures ANOVA for Performance with Group (VWD, EDD, Washout) as the between-subjects variable and evaluation Timepoint (3 mo, 6 mo, 10 mo, Final Evaluation) as the within-subjects variable with adjusted Greenhouse–Geisser degrees of freedom revealed a significant effect of Group, *F*(2, 128) = 9.423, *p* < 0.001, Timepoint *F*(2.4, 308.03) = 12.955, *p* < 0.001, and the interaction, *F*(4.81, 308.039) = 2.58, *p* = 0.028. The interaction was due to all groups improving from 3 months to 6 months and the VWDs maintaining better performance than the other groups from 10 months to the Final Evaluation, as confirmed by the following follow-up analyses. *Post hoc t*-tests revealed that scores at the 3-month were lower than at the 6-month timepoint, *p* < 0.001, VWDs scored higher than Washouts across all timepoints, *p*s < 0.01, and no difference between EDDs and Washouts, *p*s > 0.293. The VWDs scored significantly higher than EDDs at 10 months, and Final Evaluation, *p*s < 0.05.

To explore each of the Performance measures, similar separate two-way repeated-measures ANOVAs were performed on the individual measures and yielded significant main effects of Group, *p*s < 0.001, for all of the Performance measures except *Retrieve* and *Air Scenting*. Of the measures that did result in significant group differences, pairwise comparisons revealed that VWDs scored significantly higher than both EDDs and Washouts on all of the measures, with no differences between EDDs and Washouts (Table 2). The Group × Timepoint interactions were significant, *p*s < 0.05, for *Focus, Hunt, Independence*, and *Possession*; these interactions are further interpreted in Section “Timepoints.”

**Table 2:** Mean (standard error) scores for each group by measure, collapsed across time points.

		VWD	EDD	Washout
Performance	Retrieve	3.07 (0.05)	3.03 (0.11)	2.84 (0.107)
	Hunt	3.31 (0.06)^EDD,W^	2.93 (0.12)	2.87 (0.116)
	Focus	3.23 (0.06)^EDD,W^	2.84 (0.12)	2.63 (0.121)
	Possession	3.03 (0.06)^EDD,W^	2.62 (0.13)	2.67 (0.125)
	Independence	3.26 (0.06)^EDD,W^	2.95 (0.12)	2.79 (0.12)
	Work effort	3.24 (0.06)^EDD,W^	2.93 (0.115)	2.67 (0.118)
	Air scenting	3.06 (0.07)	2.79 (0.140)	2.99 (0.136)

Environmental	Surfaces	3.23 (0.05)^W^	3.22 (0.09)	3.01 (0.08)
	People	3.28 (0.06)^W^	3.16 (0.11)	2.86 (0.10)
	Vehicles	3.27 (0.05)^W^	3.13 (0.11)	2.90 (0.09)
	Visual startle	2.96 (0.10)^W^	2.98 (0.21)^W^	2.17 (0.18)
	Acoustic startle	3.13 (0.09)^W^	2.88 (0.19)^W^	2.06 (0.16)
	Excitability	2.98 (0.04)	2.92 (0.07)	2.93 (0.07)

General	Trainability	3.26 (0.05)^EDD,W^	2.82 (0.10)	2.69 (0.10)

*^EDD^Denotes that score was significantly higher than the explosives detection dog (EDD) group at the 0.05 level*.

*^W^Denotes that score was significantly higher than the Washout group at the 0.05 level*.

### Environmental Domain

Figure 2 (middle panel) shows the composite mean score for all Environmental measures across the four timepoints. A two-way repeated-measures ANOVA for Environmental with Group (VWD, EDD, Washout) as the between-subjects variable and evaluation Timepoint (3 mo, 6 mo, 10 mo, Final Evaluation) as the within-subjects variable with adjusted Greenhouse–Geisser degrees of freedom revealed a significant effect of Group, *F*(2, 131) = 8.251, *p* < 0.001, Timepoint, *F*(1.78, 233.33) = 15.30, *p* < 0.001, and the interaction, *F*(3.56, 233.33) = 4.022, *p* = 0.005. The interaction was due to generally stable scores for all groups from 3 to 6 months, and Washouts dropping significantly lower than both VWD and EDD at 10 months and Final Evaluation, as confirmed by the following follow-up analyses. VWDs and EDDs scored significantly higher than Washouts at 10 months, *p*s < 0.01, and at Final Evaluation, ps < 0.01. VWDs were equivalent to EDDs at all timepoints, *p*s > 0.36.

To explore each of the Environmental measures, similar separate two-way repeated-measures ANOVAs were conducted on the individual measures within the Environmental domain and yielded a significant main effect of Group, *p*s < 0.001, for all of the Environmental measures except *Excitability*. Of the measures that resulted in significant group differences, pairwise comparisons revealed that VWDs scored significantly higher than Washouts on all measures, but did not differ from EDDs on any measure. EDDs scored significantly higher than Washouts only on *Visual* and *Acoustic Startle* (Table 2). The Group × Timepoint interactions were significant, *p*s < 0.05, for *People* and *Vehicles*. These interactions are further interpreted in Section “Timepoints.”

### Trainability

Figure 2 (right panel) shows the mean score for the Trainability measure across the four timepoints. A two-way repeated-measures ANOVA comparing group scores for Trainability across the four timepoints with adjusted Greenhouse–Geisser degrees of freedom revealed a significant effect of Group, *F*(2, 130) = 17.218, *p* < 0.001, Timepoint, *F*(2.26, 294.57) = 6.381, *p* = 0.001, and the interaction *F*(4.532, 294.57) = 4.176, *p* = 0.002. *Post hoc* tests revealed that the VWD group had a significantly higher Trainability score than both the EDD and Washout groups, *p*s < 0.002 (see Table 2). The interaction was due to the VWDs improving across time while EDDs and Washouts decreased from 6 months to Final Evaluation, as confirmed by the following follow-up analyses. VWDs scored higher than EDDs at 10 months and Final evaluation, *p*s < 0.005, and higher than Washouts at 6 months, 10 months, and Final Evaluation, *p*s < 0.01. EDDs and Washouts did not differ at any timepoint.

### Timepoints

Independent sample *t*-tests with adjusted Levene’s test degrees of freedom were performed for each of the individual measures to determine the earliest timepoints prior to the Final Evaluation in which significant differences between groups emerged. The only measures in which group differences emerged at the 3-month timepoint were *Focus, Work Effort*, and *Surfaces*, with VWDs scoring significantly higher than Washouts on each, *p*s < 0.05.

At the 6-month timepoint, *Air Scenting* was the only measure in which VWD scored higher than EDD, *p* = 0.39, with no difference between EDDs and Washouts. VWD outperformed Washouts on *Hunt, p* = 0.02, *Focus, p* = 0.005, *Possession, p* = 0.03, *Work Effort, p* < 0.001, and Trainability, *p* = 0.008.

At 10 months, VWDs were significantly higher than EDDs on *Hunt, p* = 0.013, *Possession, p* = 0.049, *Independence, p* = 0.026, and *Trainability, p* = 0.002, and significantly higher than Washouts on every measure except *Possession, Excitability*, and *Air Scenting*.

### Sex Effects

Significantly more VWDs were male (61%) than female (39%), as confirmed by a chi-squared test of independence, *X*^2^ (1, *N* = 92) = 4.35, *p* = 0.037. Conversely, significantly more EDDs were female (80%) than male (20%), *X*^2^ (1, *N* = 25) = 0, *p* = 0.003, and no sex differences were found for the Washout group.

Separate two-way repeated-measures ANOVAs with adjusted Greenhouse–Geisser degrees of freedom were performed for each measure to determine the effect of sex (male, female), timepoint (3 mo, 6 mo, 10 mo, Final Evaluation) and their interaction. A main effect of sex was found for *Hunt, F*(1, 128) = 4.48, *p* = *0.036, Visual Startle, F*(1, 121) = 8.86, *p* = 0.003, and *Trainability F*(1, 131) = 4.541, *p* = 0.035, with males scoring higher than females. Additionally, significant interactions of Sex and Timepoint for *Hunt, F*(2.67, 342.43) = 4.78, *p* = 0.005, and *Trainability, F*(2.216, 290.361) = 3.18, *p* = 0.038, were found. Interactions between Sex and Timepoint, but no main effect of Sex, were found for *Focus, F*(2.63, 339.63) = 3.01, *p* = 0.037, *Possession, F*(2.67, 235.47) = 4.07, *p* = 0.017, *Air Scenting, F*(2.48, 247.86) = 5.10, *p* = 0.004, and *Excitability, F*(2.44, 207.77) = 4.07, *p* = 0.013.

## Discussion

The demand for dogs capable of performing increasingly specialized and challenging detection tasks is high. While dogs have been selectively bred for a variety of working tasks such as guarding, herding, and hunting for hundreds of years, the detector dog is a relatively modern development for which there has not been concerted and protracted selective breeding (3). The importance of canine detection technology in protecting against current and emerging threats establishes strong precedence for identifying, defining, and measuring behavioral characteristics in order to refine and advance canine detection capabilities.

In this study, we identified a number of behavioral characteristics that differentiate specialty VWDs suitable for detecting body-worn moving targets from standard EDDs and dogs unsuitable for service. The resulting analyses across multiple measures making up three evaluative domains, Performance, Environmental, and Trainability, provides a partial description of the VWD behavioral phenotype. Dogs were evaluated on 14 measures: seven Performance measures (characteristics related to detection and searching abilities); six Environmental measures (responses and reactions to novel and varying stimuli); and one overall Trainability measure.

### Overall Findings

Our findings further confirm the importance of behavioral characteristics as important factors in working dog suitability (1, 2, 5–9, 16–18). Analyses of individual behavioral measures suggest that, compared to standard EDDs, a number of characteristics and the degree of their expression appear to define the VWD behavioral phenotype. Furthermore, differences in search-related performance characteristics appeared to be more important than differences in environmental soundness in differentiating between VWDs and EDDs.

The partial picture of the behavioral phenotype of a VWD that emerges from the analyses of the evaluations of CPS dogs includes the following characteristics: high expression in the Performance and Trainability domains but no aggregate difference in the Environmental domain as compared to EDDs. In particular, within the Performance domain, VWDs appear to express higher overall levels of *Hunt, Focus, Possession, Independence*, and *Work Effort*, but not *Retrieve* and *Air Scenting* as compared to EDDs. However, VWDs did exhibit higher levels of *Air Scenting* at an earlier age than EDDs. At 10 months, VWDs also appeared to have greater environmental soundness in response to *Surfaces, People, Vehicles & Urban Clutter* and *Acoustic* and *Visual Startle* than Washout dogs.

A notable pattern emerging from our findings was that the majority of the Performance-related measures differentiated the VWDs from both other groups, but EDDs did not differ from Washouts in this domain. Many performance characteristics, which predominantly relate to searching and hunting behaviors, have been described in the literature as important for detector dogs. For example, detector dog handlers surveyed on their opinions of important detector dog traits identified “acuity of sense of smell” and the “tendency to hunt by smell alone” among the most important (3). Not surprisingly, then, we found that VWDs scored significantly higher on *Hunt* than both EDDs and Washouts. Interestingly, *Hunt* did not differentiate EDDs from Washouts. A likely reason for the lack of difference between EDDs and Washouts on this and all Performance measures is that the majority of Washout dogs failed due to Environmental reasons, and thus may have exhibited adequate performance-related characteristics.

Our finding that *Focus* differentiated between VWDs and EDDs is also consistent with previous reports identifying “ease of distraction” and “tendency to be distracted” as undesirable traits for working dogs (3). Sinn et al. (7) described “object focus” as an underlying dimension of military working dogs’ performance which included physical possession of objects, reflecting our finding regarding the importance of *Possession* for VWDs. Similarly, *Independence* differentiated between VWDs and EDDs, which has been commonly reported as a critical trait in a detector dog’s ability to work autonomously and not be influenced by human cueing or biasing (1, 14, 19). Dogs that are less dependent on a familiar human have also been shown to be more successful and persistent in problem-solving scenarios (20).

Perhaps the trait most widely recognized as important for detector dogs relates to an overall desire for work and is often referred to as “drive” (1, 8, 21). For example, Maejima et al. (8) found that the principal factor “Desire for Work” was associated with successful completion of training in candidate drug detection dogs. Rocznik et al. (2) also reported that operational detection dog handlers ranked search drive, the general drive to search for a hidden object, as one of the top performance characteristics for operational conditions. The incentive to search for objects out of sight is considered critical to dogs’ motivation to continue searching in strenuous conditions and contexts where the rate of encountering targets is low, as is often the case in operational contexts (1, 3, 22, 23). Consistent with this literature, *Work Effort* was a determining factor between VWDs and EDDs in our population.

Our finding that *Retrieve* did not significantly differ between groups mirrors handler rankings of this trait among the least important (3). Rocznik et al. (2) found that “chase retrieve,” the desire to pursue and pick up a thrown toy, to be marginally important to working dog handlers of different breeds. However, Slabbert and Odendaal (17) found retrieval to be an early predictor of police dog suitability. One possibility for this discrepancy may be due to breed. Dogs studied by Slabbert and Odendaal (17) were all German shepherds, whereas our study used retrievers. Given that retrievers have been bred for their propensity to retrieve objects, this trait may not vary considerably within the breed minimizing differences between individual dogs. However, our finding that a significant difference emerged at the final evaluation for *Retrieve* despite an overall effect suggests that puppy development and training may enhance this behavior in high-performing dogs.

A distinctly different pattern emerged for the Environmental domain in that VWDs did not differ from EDDs on any of these measures. While VWDs scored significantly higher than Washouts on most, EDDs only differed from Washouts on *Visual Startle* and *Acoustic Startle*. Notably, Washouts were more likely to have failed due to Environmental than Performance reasons. These findings are not surprising as fearful reactions, including reactivity to noise and novel stimuli, are widely considered undesirable traits for working dogs (24). The ability to appropriately react to, and cope with, stressful stimuli such as a variety of sights, sounds, smells, and textures, are critical for detection dogs who must work under varying conditions (1). Thus, it is likely that an environmental soundness capability threshold exists for dogs to become a detection dog of any kind, driving the lack of difference between VWDs and EDDs.

The only Environmental characteristic that did not differ between any of the groups was *Excitability*, which is found to have conflicting reports in the literature. Some instances ranked excitability lower for handler importance (3), while others rated it as one of the top measures for search team performance for operational conditions (2). Likely, the importance of excitability is operationally specific as multiple types of dog teams were evaluated in these studies. Also, as with all comparisons between such studies, definitions of the evaluative terms may differ.

Finally, Trainability scores significantly differed between VWDs and EDDs, but not between EDDs and Washouts. Trainability has been defined as the ability and speed of learning new tasks and is widely recognized as an important trait for detector dogs (1). The importance of this measure is obviously critical to a dog’s ability to learn numerous odor discriminations, corresponding behavioral responses, search patterns, and certain operational skills in as few trials and with as little direction as possible. Highly trainable dogs will reduce time and costs of training programs to produce high-quality detection dogs.

### Timepoints

VWDs were consistently highest across all four evaluation timepoints for all three domains. While VWDs showed a general increasing pattern across time in Performance and Trainability domains, EDDs and Washouts did not. Furthermore, VWDs exhibit a jump in scores for the three domains between 10 months and Final Evaluation, which coincides with the final training period, while EDDs and Washouts decrease during this time. This would suggest that the pressure imposed during final training may enhance the performance of the VWDs, while “breaking” less suitable dogs. Moreover, VWDs and EDDs Environmental scores appear generally stable over time, which likely indicates that these environmental characteristics may be more genetically determined and less influenced by experience. Washouts, however, appear to deteriorate over time on Environmental measures, with a sharp drop from 6 to 10 months. This period reflects the transition from the prison program back to CPS, which may represent a stressful event for less environmentally sound dogs. Alternatively, or perhaps in combination with, this may reflect a critical period of development which has been suggested to increase fear and awareness between 6 and 9 months (17). Evidently, service-capable dogs are better able to withstand transitions between locations. As described by Rooney et al. (24), some dogs are apparently more resilient while others are more susceptible to the same environmental disturbances.

Of significant interest to the working dog industry is the value of predicting dogs’ performance from an early age (17). Therefore, we also determined the earliest evaluation timepoints in which individual behavioral measures were predictive of success. The only measures in which groups differed at the 3-month timepoint were *Focus, Surfaces*, and *Work Effort*, in which VWDs scored higher than Washouts. At 6 months, VWDs differed from EDDs only in *Air Scenting*, but scored higher than Washouts on several other measures. Though the predictive value of early puppy tests has been questioned due to the uncertainty of the extent of environmental influence (6), “drive” or desire for work has been regarded as an innate trait that is difficult to manipulate. The finding that VWDs differed from Washouts as early as 3 months in our study may suggest a genetic basis for these particular measures. The predictability of early puppy tests may therefore only be valuable for traits with a stronger genetic basis and low susceptibility to experience. Some studies have reported high heritability of particular traits including human-directed social behavior (25), which could affect a working dogs’ focus and distractibility. Fearful behavior has also been reported to be heritable; however, without explicit genetic controls, the presence of a particular behavior cannot be determined to be inherited or environmentally influenced (24).

Few studies have reported reliable prediction of adult behavior from puppy tests and results have been mixed (26–28). Goddard and Beilharz (29) determined fearfulness was highly heritable among guide dogs and found that behavioral assessments as early as 12 weeks predicted fearfulness, with predictability strengthening at 6 months. However, evaluations of acoustic and visual startle in our study were not performed at 3 and 6 months due to the risk of creating lasting negative associations during testing (14), and so we cannot determine whether these traits may have emerged earlier. By 10 months, VWDs were significantly higher than EDDs on *Hunt, Possession, Independence*, and *Trainability*. Whether our evaluations were not sensitive enough to capture differences at earlier ages, or differences emerge due to maturity, development, training, or some combination, is not presently clear.

### Sex Differences

A sex difference was found in our population in which significantly more VWDs were male and significantly more EDDs were female. Though this may be partially attributed to a selection bias in the industry for males (1), further analyses of sex effects of individual traits revealed that overall, males scored higher than females on *Hunt, Visual Startle*, and *Trainability*, which may have contributed to overall performance. Although such differences could be affected by the bias of CPS evaluators, there is no evidence that fewer females than males were presented as candidate VWDs for sale and subject to the customer’s independent assessment. There remains the possibility that CPS employees working with young dogs are biased in the ways in which they interact with male and female dogs. However, the difference in male and female dogs may be an inherent difference in the expression of characteristics related to success as a VWD similar to biologically based sex differences seen in the expression of certain traits, such as aggression and cooperative behavior, across many species (30, 31).

In an analysis of sex differences in behavioral characteristics, Hart and Hart (32) found that males scored higher in activity levels than females. One possibility is that general activity levels may drive differences in traits related to motor activity such as *Hunt*. On the other hand, the same study also found that females ranked higher in Trainability, which is opposite to our findings. Importantly, only gonadectomized dogs were included in their study, whereas dogs in our population were left intact until point of sale; thus, inconsistencies in sex effects may be due to neuter status, which is thought to alter behavioral characteristics (32). In fact, effects of neutering on trainability have been suggested for some breeds including working dogs, indicating potential hormonal influences on this particular trait (9, 33).

Other studies have also reported effects of sex on behavioral differences specific to working dogs, though findings have been inconsistent. For example, Wilsson and Sundgren (9) found that for Labrador retrievers, “defense drive” and “hardiness” scores were higher for males than females, but females scored higher on “ability to cooperate.” Wilsson and Sundgren (28) also found increased motor activity and independence in female puppies than males. For some traits in our study, sex differences were dependent on timepoint with females scoring higher early on but lower toward the end of training, including *Focus, Possession, Air scenting*, and *Excitability*. Dogs in our population were still maturing throughout the duration of training, so males and females may have been differentially affected by developmental changes that coincided with evaluation timepoints. Another possibility reflects findings that female dogs score higher on human-directed social behavior and seek more physical contact from humans compared to male dogs, which may hinder female dogs’ working performance due to handler dependency (25).

### Overall Success of the Breeding Program

The overall success rate of the program, indicated by percentage of dogs sold as VWDs (63%) and EDDs (17%), exceeds previous reports of working dog program success rates of 30% or less (8, 9). One could argue that the overall program success rate is 80% (i.e., VW 63% + EDD 17%). VW is the standard to which dogs are bred in this program, but any dog born and raised in the program that had a final disposition of VWD or EDD can be considered a success. It should be noted that our reported success rate refers to dogs that were medically sound and does not reflect medical releases, though the number of dogs disqualified for health reasons was low (*n* = 11). Future discoveries in behavioral characterization, puppy development, and genomics may assist in elevating the success rate of detector dog breeding programs. These discoveries will help focus resources, increase the efficiency and economics of program operation, and produce adequate amounts of highly specialized dogs to detect specific targets.

### Limitations and Future Directions

Though common practice in the working dog literature, the subjective nature of behavioral evaluations is a limitation of the current study. While the aim was to have at least two independent evaluators present at each observation, this was not always possible and for practical reasons the number of evaluators and their familiarity with the dogs may have been a limiting factor. Furthermore, progress in examining characteristics across larger populations of dogs is muted by discordant definitions and procedures for scoring commonly labeled characteristics (i.e., *hunt, possession, focus, trainability, acoustic startle*, etc.) between programs. Future research should be directed at developing more objective measures of behavioral traits in order to triangulate metrics. One promising area is the use of genomics to identify genetic markers associated with behavioral phenotypes of successful detector dogs (8). Another recently advancing technology that may shed light on the neural mechanisms of behavior is the neuroimaging of awake, unrestrained canines (34). For example, Berns et al. (35) recently demonstrated the use of fMRI for predicting suitability as a service dog, and investigations by Auburn University’s multidisciplinary *Canine fMRI Team* have shown that canine brain response to trained odors (36) and brain connectivity patterns and their strengths are related to behavioral assessments of working dog performance (37).

Comparisons between VWD, EDD, and Washout dogs at each timepoint were conducted in order to determine whether differences between groups emerged at early ages. Early prediction of such differences would allow for the efficiency of redirecting dogs unlikely to be successful as detector dogs to other purposes at an early age. Although we found some measures to differentiate successful vs. unsuccessful candidates as early as 3 months, the relative contributions of inherited characteristics, maturation, and past experiences cannot be isolated.

It should also be acknowledged that our use of final disposition at point of sale as group determination may not necessarily be a reliable indication of continued long-term service. Though many studies have used program outcome as classification of success for working dogs, few have followed up to determine the longevity of such classifications. One study with guide dogs found low retention 1 year after program graduation, with significantly more dogs successfully completing their training program than were still working 1 year later (38). Though we did not obtain data on long-term success of dogs in our program, the 30-day post-sale window in which vendors were able to return dogs increases the validity of our endpoint, to some extent, compared to sale status at the completion of training alone. Future studies should track the continued success of working dogs well into their service.

We have recently begun collecting measurements of the following additional behavioral characteristics that we believe may help further refine the VWD phenotype (not included in the current sample). *Engagement*: Extent to which a dog is eager to please and involve the handler in its execution of a directed task, remaining involved in the game and returning rewards to handler to engage in play. This characteristic has been added because we have produced some dogs with an extreme propensity to search for odors but with very low interest in a reward object or interaction with a handler, which interferes with the handler’s management of the working of the dog. *Hypervigilance*: Excessive attention to the environment due to apprehension of potential threats—exhibits anxiety/fear, repeatedly scans environment, overly responsive and cowers in response to mild-moderate visual and auditory stimuli. *Distractibility*: Extent to which ongoing searching is interrupted by attention and/or attraction (not fearful or anxious) to objects, people, or other activities occurring in the environment—execution of task easily or frequently interrupted by ancillary events in surroundings, differentiated from “focus,” the measurement of which is mostly related to attending to reward or immediate presence of odor, by measurement during operational style searches. Additionally, age-appropriate acoustic and visual startle tests have been adapted for 3- and 6-month-old puppies in order to examine how such reactivity may predict the environmental soundness and/or success earlier than 10 months of age for VWDs and EDDs.

## Conclusion

Search-related performance traits appear to be critical factors that elevate a detector dog from standard EDD suitability to VWD quality. On the other hand, certain traits related to environmental soundness appear to be important for a detector dog of any kind, differentiating both VWDs and EDDs from Washouts. Since 2013, CPS has produced 63% VWDs and an additional 17% EDDs from its breeding program suggesting that selective pressure has amplified behavioral characteristics that support VWD and EDD performance.

This work represents the first examination of the expression behavioral characteristics related to the success of *Vapor Wake*^®^ detection, a specialized application for detecting body-worn or hand-carried explosives in settings with large volumes of moving persons, such as large event venues and mass transit. As such, this study is also one of the first to identify specific characteristics for any specialized detector dog application. The specialization and sophistication of detector dog applications is necessarily increasing to meet modern security and safety requirements. Identifying the characteristics associated with success in the performance of specialized detector dog applications will be critical to producing the necessary numbers and quality of dogs to fulfill future security and safety needs.

Identification, measurement, and validation of the contribution of particular behavioral characteristics to performing the VW task is vital to driving selective breeding and possible future genotyping for continual improvement of dogs for this task. Such phenotyping efforts support the tailored design of detector dogs for specialized applications, which are becoming more prevalent in response to the need for enhanced uses of dogs in security and safety operations. Although this present work is specific to VWDs, whether within or outside of the CPS breeding cohort, it is an example of a more general strategy to enhance the identification and production of dogs for specialized applications. If refined and practiced on a large scale, it could be envisioned that populations of purpose-bred dogs with highly defined behavioral phenotypes and identified genetic markers for particular characteristics might exist from which to build evermore technically competent detector dogs for specialized applications.

## Ethics Statement

Dog care and use activities were approved and monitored by the Auburn University Institutional Animal Care and Use Committee.

## Author Contributions

LL designed the study, analyzed data, and primarily prepared the manuscript. PH conducted selective breeding activities, collected data, and contributed to design of the study. PH, JB, TF, BR, and PW designed the evaluative instruments. JB, TF, and BR performed evaluations. PH and PW contributed to the preparation of the manuscript. CA, JK, and PW supervised the design of the study and preparation of the manuscript. JK supervised analyses of the data.

## Conflict of Interest Statement

PW and JB are among the inventors of VW technology who receive a portion of the royalties collected by Auburn University upon the sale of VWDs by the licensee of VW technology. Otherwise, the authors declare that the research was conducted in the absence of any commercial or financial relationships that could be construed as a potential conflict of interest.
